# Determinants of cancer screening intentions among UAE residents: an exploratory model with application of the information-motivation-behavioral skills model

**DOI:** 10.3389/fpubh.2026.1912283

**Published:** 2026-10-02

**Authors:** Ramya Kundayi Ravi, Mona Gamal Mohamed, Sasidharan Sivakumar, Nilda Ogsimer

**Affiliations:** 1 RAK College of Nursing, RAK Medical and Health Sciences University, Ras Al Khaimah, United Arab Emirates; 2 Discovery Research Division, Indian Council of Medical Research, ICMR Headquarters, Ansari, New Delhi, India

**Keywords:** Cancer, intention, motivational, perceptions, screening, structural equation modeling, United Arab Emirates

## Abstract

**Introduction:**

Cancer remains a leading cause of morbidity and mortality worldwide, making the promotion of early detection through cancer screening a major public health priority. Understanding the factors that influence individuals’ intentions to participate in cancer screening is essential for developing effective interventions and policies aimed at improving screening uptake. This study examined the determinants of cancer screening intention among residents of the United Arab Emirates (UAE) using the information–motivation–behavioral skills (IMB) model as a theoretical framework.

**Methods:**

A cross-sectional study was conducted among 239 UAE residents recruited from outpatient departments. Data were collected using a structured questionnaire developed based on a modified IMB model. The questionnaire assessed sociodemographic characteristics, knowledge regarding cancer and cancer screening, attitudes toward screening, behavioral skills related to screening, and cancer screening intention. Cancer screening intention was assessed using a single dichotomous item. Descriptive statistics were performed using JASP software, while confirmatory factor analysis (CFA) and latent variable structural equation modeling (LV-SEM) were conducted using Onyx software to examine the relationships among observed and latent variables.

**Results:**

The structural equation model demonstrated relatively poor fit to the data (*χ*^2^/df = 4.34, CFI = 0.824, TLI = 0.801, RMSEA = 0.118), and the findings should therefore be interpreted as exploratory. Family history of cancer (*β* = 0.39, *p* < 0.001), perceived personal risk of cancer (*β* = 0.30, *p* < 0.001), and participation in cancer awareness workshops (*β* = 0.16, *p* < 0.01) emerged as significant positive predictors of intention to undergo cancer screening. Attitudes toward cancer screening were positively associated with behavioral skills (*β* = 0.33, *p* < 0.001). In contrast, knowledge about cancer (*β* = −0.01, *p* = 0.264) and knowledge about cancer screening (*β* = −0.20, *p* = 0.133) were not significantly associated with behavioral skills. Furthermore, the hypothesized mediating role of behavioral skills was not supported.

**Discussion:**

The findings suggest that perceived susceptibility, familial experience with cancer, and engagement in awareness activities are more influential determinants of screening intention than knowledge alone. Interventions aimed at improving screening uptake should emphasize personalized risk communication, family-centered approaches, and culturally tailored strategies that address motivational and practical barriers. Further research using longitudinal and nationally representative designs is needed to validate these findings and refine theoretical models of cancer screening behavior in the UAE.

## 1 Introduction

Cancer is the second leading cause of mortality worldwide. Nearly one in six deaths, approximately 10 million deaths were attributed to cancer in 2020 (1). Projections indicate that the burden from cancer is projected to increase exponentially by more than 60% by 2040 with the growth and aging of the population if measures are not taken to curb its incidence and resultant mortality (2, 3).

Cancer is a major public health threat in the United Arab Emirates (UAE) as well (4). According to recent data, the age-standardized cancer incidence rates in the UAE are 70.1 per 100,000 for males and 74.2 per 100,000 for females. (5). The country has included reducing the number of deaths due to cancer as one of the key performance indicators in the National Agenda and is on devising plans and policies at all levels of health care to curb the incidence of cancers and build a health system that facilitates access to timely quality diagnosis and treatment (6). Despite healthcare facilities in many Emirates meeting international standards and the availability of screening programs, a comprehensive national cancer control plan has not been implemented across the country (6).

Recent studies in Middle Eastern populations have applied established behavioral frameworks to understand cancer screening. Studies reported from Saudi Arabia and Jordan have used the Health Belief Model to examine factors associated with cervical and lung cancer screening, highlighting the importance of perceived susceptibility, benefits, barriers, and other health beliefs (7, 8). Similarly, recent research from Iran has applied the Theory of Planned Behavior to examine cognitive factors associated with cancer screening uptake, emphasizing attitudes, subjective norms, perceived behavioral control, and behavioral intention (9, 10).

The Information–Motivation–Behavioral Skills (IMB) model provides a framework for understanding health-related behaviors by integrating three interrelated domains: information, motivation, and behavioral skills. Unlike models that primarily emphasize health beliefs or attitudes and perceived behavioral control, the IMB framework considers whether individuals possess sufficient information, motivation, and skills to initiate and maintain health-related behaviors (11). Recent studies have demonstrated the applicability of the IMB framework to cancer screening, including research among Iranian women and working populations in China (12).

Despite increasing evidence on behavioral determinants of cancer screening in Middle Eastern populations, limited evidence has examined cancer screening intention among UAE residents using an integrated framework encompassing information, motivation, and behavioral skills. Moreover, although HBM and TPB have been widely applied to cancer screening, relatively little is known about whether the IMB framework can explain screening intention within the sociocultural and healthcare context of the UAE. Therefore, this study applied the IMB model to examine the relationships among cancer-related information, motivation, behavioral skills, and cancer screening intention among UAE residents.

Diagnosis and management of cancer have a huge impact on the physical, emotional, social, and economic well-being on individuals and their family members (13). Cancer care and its complications also enforce a marked economic burden on all countries from the loss of productivity from early morbidity and premature mortality, and increased healthcare spending (14). In addition, reallocating resources wisely for individual and population-based programs can bring down the future catastrophic economic burden on sustainable economic and human development. Recent analysis shows that investing in screening, cancer diagnostics, and treatment services can modalities, advanced imaging modalities could yield substantial health and economic benefits up to 2.9 trillion USD in a lifetime (15).

In 2017, the World Health Organization (WHO) urged policymakers and program managers to develop and implement feasible and cost-effective programs for the early diagnosis and screening of cancer. There is evidence that delay in the diagnosis and treatment of cancer is associated with poor quality of life, higher economic cost, and increased mortality. Hence it is of paramount importance that appropriate screening programs are developed, communicated and implemented at individual and population levels for the early diagnosis and appropriate treatment of cancer (16).

## 2 Conceptual framework

This study is grounded in the information-motivation-behavioral skills (IMB) theory and model, developed by Jeffrey D. Fisher and William A., which synthesizes constructs from multiple health behavior theories (17). The IMB model comprises three core components: information, motivation, and behavioral skills. It posits that individuals must be well-informed, motivated both internally and externally, and equipped with the necessary behavioral skills to adopt, implement, and sustain positive health behaviors (17). This framework has been successfully applied across diverse populations to predict outcomes such as HIV risk and prevention (18), disease prevention (19) screening uptake (20), treatment adherence (21) resilience and quality of life improvement, (22) and the promotion of health behaviors (23).

The current study aims to (1) assess the applicability of the IMB model in predicting the intention to undergo cancer screening and (2) elucidate the interrelationships among awareness (information), motivation (internal and external), and behavioral skills that influence screening behavior. These insights will inform targeted recommendations to enhance cancer screening rates within the study population.

Guided by established theoretical frameworks, two categories of hypotheses-direct effects and moderating effects-were developed for this exploratory analysis. The conceptual framework incorporates key latent variables, including knowledge about cancer, knowledge regarding cancer screening, and attitudes toward cancer screening. These latent constructs were operationalized through a series of observed variables designed to capture their underlying dimensions. The analysis first examined the direct effects of these latent variables on the primary outcome variable: the intention to undergo cancer screening.

Beyond these direct effects, the framework further investigates the mediating role of behavioral skills in the relationship between knowledge, attitudes, and screening intention. Specifically, it explores how knowledge and attitudes may indirectly influence the intention to screen by enhancing behavioral skills relevant to cancer screening. Additionally, the model integrates critical contextual factors such as perceived cancer risk, family history of cancer, and prior participation in cancer-related workshops. These variables were assessed both independently and within the broader latent variable context to determine their influence on screening intention.

This comprehensive conceptual framework thus facilitates a nuanced understanding of the multifaceted drivers of cancer screening intention, supporting the formulation of evidence-based recommendations to promote screening behavior within the target population.

The following are the hypothesis developed and tested as a part of the study.

H1: Knowledge about cancer positively influences behavioral skills related to cancer screening.

H2: Attitude towards cancer screening positively influences behavioral skills.

H3: Behavioral skills mediate the relationship between knowledge and attitudes towards cancer screening and the intention to undergo cancer screening.

H4: Family history of cancer is positively associated with the intention to undergo cancer screening.

H5: Perceived risk of cancer is positively associated with the intention to undergo cancer screening.

H6: Participation in cancer awareness workshops or campaigns is positively associated with the intention to undergo cancer screening.

## 3 Methodology

### 3.1 Design and setting

The study, conducted between September 2022 and December 2023, utilized a cross-sectional, exploratory survey design. The study followed the STROBE reporting guidelines. The participants were recruited from the outpatient departments of two selected hospital, from the Northern Emirate of Ras Al Khaimah, United Arab Emirates.

### 3.2 Sample and sample size

The target population comprised all residents of the Emirate of Ras Al Khaimah, United Arab Emirates (UAE), aged 18 years and older. Participants were required to be proficient in either English or Arabic and living in Ras Al Khaimah for the past 6 months. Those were unable to provide adequate responses due to physical or cognitive impairments, as well as those with a history of cancer, were excluded from the study.

A convenience sampling method was employed to recruit participants. The sample size was determined based on structural equation modeling requirements, considering the complexity of the proposed model and the number of estimated parameters. With 239 participants, the study exceeded the commonly recommended minimum sample size of 200 for SEM and met the suggested participant-to-parameter ratio of at least 10:1, supporting stable parameter estimation and adequate model testing (24, 25).

### 3.3 Measurement and scoring

#### 3.3.1 Latent and observed variables

Prevalidated, and reliable questionnaire developed by the investigators was used for data collection. The questionnaire utilized in this study was constructed based on a comprehensive review of pertinent literature and established research (26, 27). Following the establishment of content validity (CVI = 0.92) and reliability (*r* = 0.87), the instrument was translated into Arabic, the local language. A subsequent back-translation into English was conducted to ensure accuracy and consistency. The final version of the questionnaire had five sections.

*Section 1-Participant characteristics*: This section gathered demographic and personal information from participants using 14 structured, closed-ended questions. The questions covered various aspects, including age, gender, marital status, and family history of cancer, among others.

Perceived risk of cancer: This section explored participants’ self-assessed risk of developing cancer. Participants were asked to quantify their perceived risk on a numerical scale ranging from 0 to 10, where 0 represents “not at all at risk” and 10 signifies “very high risk.”

*Section 2-Information*: Information was measured by the knowledge of cancer warning signs and symptoms, risk factors of cancer, and information regarding cancer screening. A 24-item trichotomous scale (Yes = 1, No = 0, do not know = 0) measured knowledge of warning signs and symptoms (9 items) and risk factors (15 items). Awareness of cancer screening was evaluated using a five-point Likert scale (ranging from “not at all aware” to “extremely aware”) comprising five items, with scores ranging from 1 (“not at all aware”) to 5 (“extremely aware”). Negatively worded items were reverse-coded. Total scores were calculated by summing individual item scores, resulting in a range of 0–24 for cancer knowledge and 5–25 for screening awareness.

*Section 3-Motivation*: Motivation towards cancer screening was evaluated using a 10-item, five-point Likert scale, encompassing eight items on attitude and two items on social norms. Responses ranged from 1 (“strongly disagree”) to 5 (“strongly agree”). Negatively worded items were reverse-coded. Total scores were calculated by summing individual item scores, yielding a potential range of 10–50.

*Section 4-Behavioral skills*: Behavioral skills related to cancer screening were assessed using an eight-item, five-point Likert scale, encompassing four items on self-efficacy and four items on objective ability. Responses ranged from 1 (“strongly disagree”) to 5 (“strongly agree”). Negatively worded items were reverse-coded. Total scores were calculated by summing individual item scores, resulting in a potential range of 8–40.

*Section 5-Screening intention*: Intention to undergo cancer screening was measured using a single-item question: “Are you willing to participate in cancer screening in the future?” Response options were dichotomous: 1 = No and 2 = Yes.

Participants were asked a single dichotomous question regarding their willingness to undergo cancer screening in the future. Importantly, the survey did not define ‘cancer screening’ beyond the general term, nor did it specify particular screening modalities (e.g., mammography, colonoscopy). Therefore, participants’ responses reflect their general inclination toward screening as they individually interpreted the term, rather than intention toward any specific test.

### 3.4 Data collection

The questionnaire, designed specifically for the study, was administered in a paper-based format by the research team. Eligible participants were approached by the investigators, who provided a detailed explanation of the study’s objectives, purpose, and duration. Participants were informed of the anonymous and voluntary nature of the survey and assured they could withdraw at any time without consequence. A participant information sheet and consent form were provided in paper format, and only those who consented were enrolled in the study. Completion of the questionnaire took approximately 12 to 15 min, and no issues were encountered during recruitment or data collection. All data were collected anonymously, with no personal identifiers, and participants did not receive any form of compensation.

A pilot study involving 20 participants was conducted to assess the feasibility of the study procedures. The findings indicated that the study was feasible, and no modifications were required to the study protocol, data collection procedures, or analysis plan. The pilot study also helped finalize the methods for data collection and analysis. Data obtained during the pilot phase were not included in the final analysis.

### 3.5 Statistical analysis

The data gathered were organized using Microsoft Excel, and further descriptive statistics, including frequency, percentage, mean, and standard deviation for all variables, were calculated with JASP software. To examine the causal relationships between observed and latent variables, confirmatory factor analysis and latent variable structural equation modeling were performed using Onyx software. The model’s fit was evaluated through several indices that are typically used in structural equation modeling (SEM) analysis. The process of reaching this near-fit model involved meticulous refinement through the iterative removal of variables at three stages of observation; (1) Items with factor loadings less than 0.40 were removed to improve the model’s accuracy, (2) Parameters with high residual variances (greater than 0.50) were identified and removed, as they indicated poor fit within the model, (3) Indicators with low R^2^ values (below 0.50) were excluded, as they did not adequately represent the latent constructs (28). The model was considered acceptable for further analysis only if it met the recommended criteria; goodness-of-fit (GOF) measures, which include the chi-squared fit statistic (*χ*^2^/df), root-mean-square error of approximation (RMSEA), goodness-of-fit index (GFI), Tucker-Lewis Index (TLI), and comparative fit index (CFI) (29). Acceptable model fit was indicated when these indices approached or exceeded recommended levels. The ratio, *χ*^2^/pdf of ≤3, CFI of >0.90, and RMSEA of <0.08 indicated an acceptable model fit (30).

Maximum likelihood estimations were employed in a latent variable SEM analysis to examine the causal links between observed and latent variables in the model. This method ensures that the conclusions drawn are based on dependable and valid statistical inferences, which ultimately aids in making better clinical decisions and enhancing patient outcomes (31).

## 4 Results

### 4.1 Profile of participants

Most participants were female (52.7%), Emirati (61.9%), married (81.6%), and had a college education or higher (95.4%). The largest age group was 31–40 years (42.7%), and most were employed in the private sector (72.8%). While the majority had no family history of cancer (63.2%), nearly two-thirds expressed an intention to undergo cancer screening (64.4%), indicating a generally positive inclination toward screening (Table 1).

**Table 1:** Participant profile.

Variables	Categories	*n*	Percentage
Age (years)	18–30	91	38.1
	31–40	102	42.7
	>41	46	19.2
Gender	Male	113	47.3
	Female	126	52.7
Nationality	Emirati	148	61.9
	Non-Emirati	91	38.1
Marital status	Married	195	81.6
	Single	44	18.4
Educational level	Secondary school	11	4.6
	College & above	228	95.4
Occupation	Private employee	174	72.8
	Government employee	14	5.9
	Merchant	2	0.8
	Unemployed	49	20.5
Presence of chronic illness (self-reported)	Nil	151	63.2
	≤2	82	34.3
	≥2	6	2.5
Family history of cancer	Yes	149	62.3
	No	90	37.7
Habit of smoking	No	218	91.2
	Yes	21	8.8
The habit of alcohol consumption	No	85	35.6
	Yes	154	64.4

### 4.2 Confirmatory factor analysis

#### 4.2.1 The IMB model estimation

The Information-Motivation-Behavioral (IMB) model components were rigorously assessed using Confirmatory Factor Analysis (CFA). The model is shown in Figure 1. The assessed model fit indices suggest that the model is approaching an acceptable fit; Chi-Square test from the baseline model (*Χ*^2^ = 4508.963, df = 253), chi-square test to the factor model (*Χ*^2^ = 972.860, df = 224, *p* < 0.001), comparative Fit Index (CFI): 0.824, Tucker-Lewis Index (TLI): 0.801, Root Mean Square Error of Approximation (RMSEA): 0.118, Standardized Root Mean Square Residual (SRMR): 0.070, Goodness of Fit Index (GFI): 0.934. The structural equation model demonstrated a marginal fit to the data, and findings should therefore be interpreted as exploratory. (*χ*^2^/df = 4.34, CFI = 0.824, TLI = 0.801, RMSEA = 0.118). This refined model now provides a solid foundation for subsequent model analysis, ensuring its theoretical soundness and practical feasibility.

**Figure 1:**
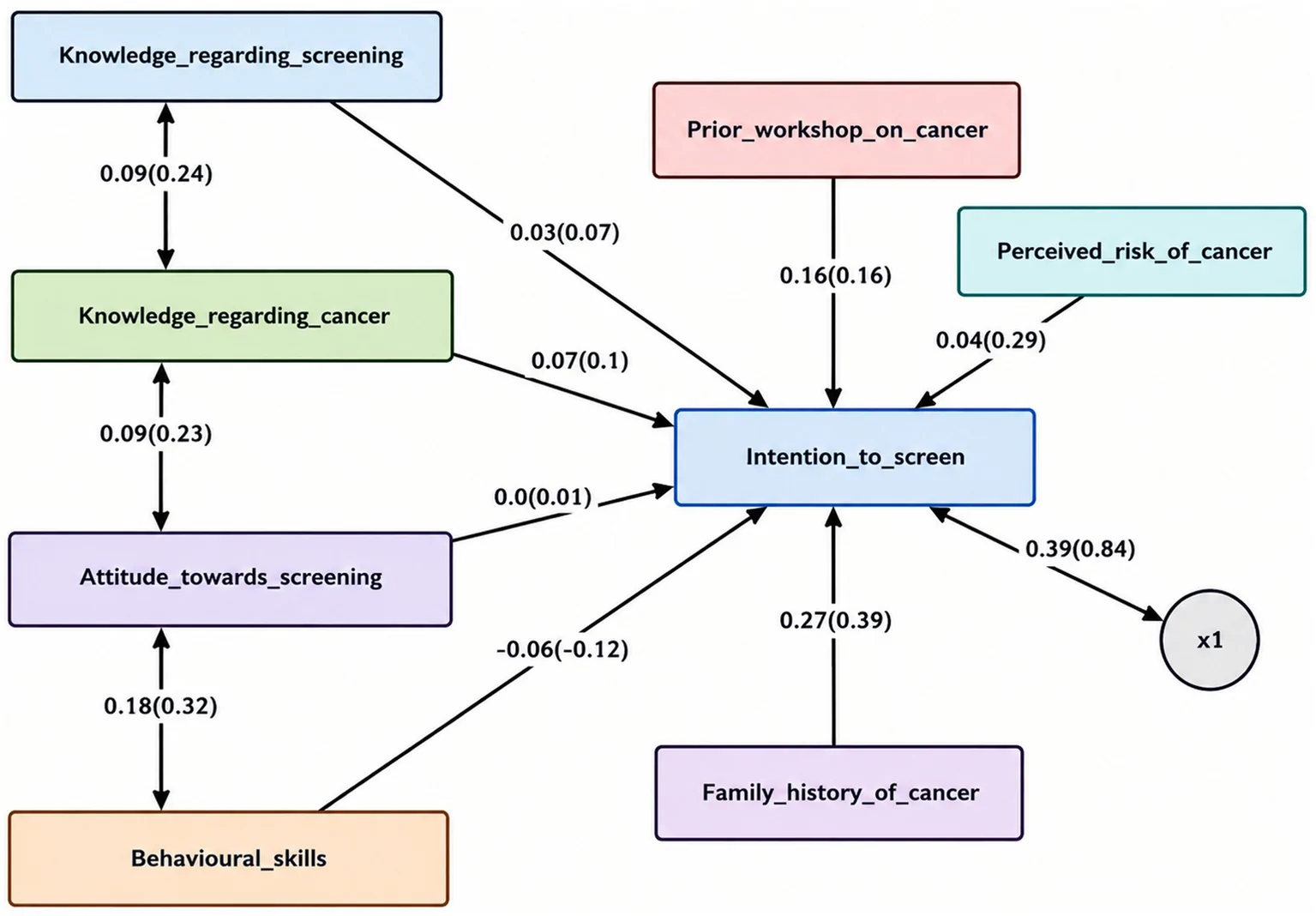
Latent variable structural equation model.

Table 2 presents the estimated parameters for the observed variables, including significant factor loadings, standard errors, and *R*^2^ values, as derived from the Confirmatory Factor Analysis (CFA) demonstrating the validity and reliability of the observed variables in representing the latent constructs.

**Table 2:** Standardized estimated factor loadings, standard errors, and *R*^2^ values of the IMB model constructs of latent variables.

Observed variables	Estimate	Standard error	*Z* value	*R* square	*p* value
Knowledge regarding signs, symptoms, and risk factors (information)					
An unexplained lump or swelling	0.335	0.028	11.981	0.478	<0.001
An unexplained bleeding	0.396	0.033	12.097	0.484	<0.001
A persistent cough or hoarseness of voice	0.47	0.034	13.877	0.588	<0.001
A persistent change in bowel or bladder habits	0.517	0.033	15.832	0.701	<0.001
Persistent difficulty in swallowing	0.562	0.032	17.453	0.791	<0.001
A change in the appearance of a mole	0.597	0.033	17.862	0.812	<0.001
A sore that does not heal	0.52	0.034	15.092	0.659	<0.001
An unexplained weight loss	0.476	0.032	14.798	0.643	<0.001
Excessive exposure to sunlight	0.356	0.032	11.118	0.424	<0.001
Frequent consumption of red or processed meat	0.401	0.037	10.990	0.416	<0.001
Increased consumption of sugar	0.399	0.036	11.100	0.423	<0.001
Family history of cancer	0.346	0.029	12.132	0.484	<0.001
Knowledge regarding cancer screening (information)					
Awareness regarding the benefits of cancer screening	0.608	0.047	13.022	0.540	<0.001
Awareness regarding side effects of cancer screening	0.791	0.046	17.116	0.772	<0.001
Awareness regarding kinds and intervals of cancer screening	0.834	0.044	19.046	0.874	<0.001
Awareness regarding cancer screening programs available in the country	0.839	0.045	18.767	0.860	<0.001
Awareness regarding financial support systems for patients diagnosed with cancer	0.809	0.046	17.427	0.789	<0.001
Attitude towards screening (motivation)					
It is difficult for me to arrange transport to the visit the doctor	0.862	0.060	15.398	0.891	<0.001
I am not confident to talk about my symptoms with the doctor	0.729	0.062	11.750	0.550	<0.001
I am embarrassed to undergo cancer screening	0.463	0.073	6.311	0.194	<0.001
Behavioral skills					
Ability to endure psychological troubles during the examination	0.792	0.058	13.736	0.743	<0.001
Ability to endure the fear related to the results of the cancer screening	0.687	0.055	12.441	0.600	<0.001
Easy to register for cancer screening and to check its results.	0.373	0.047	7.874	0.286	<0.001

*p*-value of ≤0.05 is considered significant.

#### 4.2.2 Reliability of the latent variable constructs

The internal consistency and reliability of the latent variables in the confirmatory factor analysis were assessed using both Coefficient *ω* and Coefficient *α*. The results indicated high reliability across the variables, with ‘Knowledge on cancer’ and ‘Knowledge of screening’ demonstrating the strongest internal consistency, evidenced by coefficients of *ω* = 0.939 and 0.944, and *α* = 0.941 for both. The ‘Attitude towards screening’ variable exhibited moderate reliability with *ω* = 0.753 and *α* = 0.748, while ‘Behavioral skills’ showed good reliability with *ω* = 0.800 and *α* = 0.760. The overall reliability across all variables was robust, with a total Coefficient *ω* of 0.920 and Coefficient *α* of 0.870, indicating that the constructs measured are both reliable and consistent for further exploratory analysis.

#### 4.2.3 Correlation between the latent variables in the IMB model

Table 3 indicates a significant positive correlation between the latent variables “knowledge regarding cancer” and both “knowledge about cancer screening” and “attitude towards cancer screening.” This suggests that participants with higher knowledge about cancer are likely to have better knowledge about screening and a more positive attitude towards screening. However, the analysis revealed that the other latent variables were not significantly correlated with each other, indicating that their relationships may be independent or less influential within the model.

**Table 3:** Factor covariances between the latent variables.

Latent variables	Estimate	95% confidence interval		*z*-value	*p* value
		Lower	Upper		
Knowledge on cancer ↔ Knowledge on screening	0.262	0.138	0.386	4.133	< 0.001
Knowledge of cancer ↔ Attitude towards screening	0.236	0.108	0.365	3.603	< 0.001
Knowledge of cancer ↔ Behavioural skills	0.082	−0.062	0.225	1.117	0.264
Knowledge of screening ↔ Attitude toward screening	0.099	−0.033	0.23	1.475	0.14
Knowledge of screening ↔ Behavioural skills	−0.111	−0.255	0.034	−1.502	0.133
Attitude towards screening ↔ Behavioural skills	0.393	0.268	0.518	6.156	< 0.001

A *p*-value of ≤0.05 is considered significant.

### 4.3 Mediation analysis

The mediation analysis reveals the complex relationships between the latent, observed, and dependent variable, intention towards screening. This analysis specifically examines the mediating effects of the knowledge regarding cancer, knowledge, and attitude on screening, family history of cancer, prior workshops related to cancer, and the perceived risk of cancer with the intention to screen through the mediating variable behavioral skills. The results indicate that the relationship between these variables and the intention to screen is generally weak, as none of the estimated variables demonstrated a statistically significant mediating effect (Table 4).

**Table 4:** Estimates from mediation analysis.

Variables	Estimate	Std. error	*z*-value	*p*
Knowledge regarding cancer→ Behavioural skills→ Intention to screen	0.001	0.007	0.158	0.87
Knowledge regarding screening→ Behavioural skills→ Intention to screen	0.01	0.006	1.67	0.09
Attitude towards screening → Behavioural skills→ Intention to screen	−0.016	0.008	−1.846	0.06
Family history of cancer→ Behavioural skills→ Intention to screen	−0.009	0.008	−1.184	0.23
Prior workshop on cancer→ Behavioural skills→ Intention to screen	0.01	0.009	1.086	0.27
Perceived risk of cancer→ Behavioural skills→ Intention to screen	0.002	0.001	1.235	0.21

*p* value of ≤0.05 is considered significant.

### 4.4 Exploratory latent variable structural equation modelling (SEM) analysis

Figure 1 presents a detailed overview of the factors influencing participant’s intentions to undergo cancer screening, analyzed using the Information-Motivation-Behavioral Skills (IMB) model through exploratory Structural Equation Modelling (SEM). The measurement model demonstrated significant covariances among independent variables, which were consistent in the final SEM analysis. Among the variables influencing cancer screening intentions, the observed variable, prior family history of cancer exhibited the highest factor loading (0.39), indicating its substantial impact. This was followed by a subjective perceived risk of cancer, with a factor loading of 0.29. These findings suggest that subjective perceptions, such as the personal fear of developing cancer, have a more pronounced effect on screening intentions than objective knowledge about cancer or screening processes. Additionally, exposure to prior cancer-related workshops had a factor loading of 0.16, further underscoring the role of experience-based influences on intention to screen.

Among the latent variables, knowledge of cancer signs showed a factor loading of 0.10. Although lower than the direct observed variables, it was the most influential among the latent factors assessed. An inverse association was observed between behavioral skills related to cancer screening and screening intention. This finding was contrary to the hypothesized positive relationship within the IMB framework and was therefore interpreted cautiously given the exploratory nature and relatively poor fit of the overall model.

## 5 Discussion

This study employed the Information-Motivation-Behavioral Skills (IMB) model to explore the determinants of cancer screening intentions among residents of the United Arab Emirates. The study participants were predominantly middle-aged adults and Emiratis, reflecting a representative population. In the exploratory SEM, family history of cancer (*β* = 0.39, *p* < 0.001), perceived personal risk of cancer (*β* = 0.30, *p* < 0.001), and participation in cancer awareness workshops (*β* = 0.16, *p* < 0.01) showed positive associations with cancer screening intention. Given the relatively poor overall model fit (*χ*^2^/df = 4.34, CFI = 0.824, TLI = 0.801, RMSEA = 0.118), these associations should be interpreted as exploratory rather than as definitive confirmation of the hypothesized theoretical relationships. Notably, knowledge about cancer and cancer screening were not significantly associated with behavioral skills, suggesting that information alone may not translate into screening readiness in this population. In contrast, positive attitudes towards screening showed a strong positive relationship with behavioral skills (*β* = 0.33, *p* < 0.001), supporting the motivational component of the IMB model.

In the Information component, we examined three key areas: warning signs and symptoms, risk factors of cancer, and cancer screening awareness. The warning signs and symptoms showed varying levels of recognition among participants. “A change in the appearance of a mole” had the highest factor loading (0.597), followed closely by “A persistent difficulty in swallowing” (0.562) and “A persistent change in bowel or bladder habits” (0.517). These findings suggest that visual changes and persistent physical symptoms are more readily recognized as potential cancer indicators by UAE residents. However, “An unexplained lump or swelling,” often considered a classic cancer symptom, had the lowest factor loading (0.335). This difference is concerning and differs from findings in Western populations, where lumps are typically highly recognized as a cancer symptom (32, 33). This result highlights a critical area for targeted public health education in the UAE. Consistent with our findings, a study conducted in the UAE revealed that nearly half of the women participated in the study had never undergone a Clinical Breast Exam or mammogram. Additionally, many participants misunderstood the significance of a breast lump and showed less concern about its seriousness than expected (34).

Regarding risk factors of cancer, the factor loadings were generally lower than those for symptoms. “Frequent consumption of red or processed meat” (0.401) and “Increased consumption of sugar” (0.399) had the highest loadings, while “Family history of cancer” had a surprisingly low loading (0.346). These findings contrast with studies in other populations, such as Wang et al. in the China, where family history is often well-recognized as a significant risk factor (35). The observed lack of awareness regarding family history as a significant risk factor for cancer among participants in our United Arab Emirates (UAE) study represents a crucial area that requires targeted attention in upcoming public health initiatives.

The variables related to cancer screening awareness demonstrated markedly strong factor loadings in our study, particularly for “Awareness regarding cancer screening programs available in the country” (0.839) and “Awareness regarding the types and recommended intervals for cancer screening” (0.834). These findings indicate that initiatives aimed at increasing public knowledge about cancer screening options in the UAE have been largely effective. However, despite this high awareness, the lack of significant association between knowledge of screening and behavioral skills (*p* = 0.133) reveals a critical gap between awareness and action. In alignment with our results, Sheshachalam et al. and Wang et al. discovered that both individuals’ confidence in their perceived cancer awareness and their actual level of awareness were relatively low. They found that only those with postgraduate degrees displayed a moderate understanding of certain details related to cancer epidemiology, indicating that higher educational attainment is linked to a somewhat better comprehension of these nuances (36, 37). Also, in the line study a study conducted by Alsowiyan et al. revealed that the overall knowledge of breast cancer among women was insufficient. Although approximately 50% of the participants reported regularly conducting breast self-examinations, the rate of clinical breast examinations was lower, indicating a disparity in breast examination practices (38).

In the Motivation component, there is a prominent contrast between different barriers to screening. “It is difficult for me to arrange transport to visit the doctor” had an exceptionally high factor loading (0.862), indicating that logistical barriers are a major concern for UAE residents. This finding aligns with Hallgren et al. in the USA who have identified practical barriers as significant deterrents to cancer screening, especially the transportation (39). Conversely, “I am embarrassed to undergo cancer screening” had a much lower loading (0.463), suggesting that emotional or cultural barriers, while present, may be less prominent than practical obstacles. A previous study conducted in Saudi Arabia on cancer screening identified fear of the screening procedure itself and anxiety about the potential results as significant barriers to screening participation among the study population (40).

The Behavioral Skills component revealed interesting patterns. “Ability to endure psychological troubles during the examination” had the highest loading (0.792), followed by “Ability to endure the fear related to the results of the cancer screening” (0.687). These high loadings suggest that psychological resilience plays a crucial role in individuals’ perceived ability to undergo cancer screening. “Easy to register for cancer screening and to check its results” had a much lower loading (0.373), indicating that practical aspects of the screening process may be perceived as less challenging than the emotional aspects. Supporting our data, a study by Howell et al. also demonstrated that fear of screening results significantly impacts individuals’ intention and motivation to undergo cancer screening (41). This highlights the critical need for healthcare providers to address and alleviate these fears through effective patient education and support, to improve screening rates and early detection of cancer.

The most significant predictors of screening intentions in our study were family history of cancer (*β* = 0.39, *p* < 0.001) and perceived personal cancer risk (*β* = 0.30, *p* < 0.001). Participation in cancer awareness workshops also showed a significant positive influence (*β* = 0.16, *p* < 0.01). These findings align with the Health Belief Model by Karl et al. (42) are consistent with studies in other populations. For instance, a meta-analysis by Feng et al. (43) found that perceived risk was a strong predictor of cancer screening behavior across various cultural contexts. The strong influence of these factors in our UAE sample suggests that risk-based screening promotion could be particularly effective in this population. Public health campaigns emphasizing personal risk assessment and the importance of screening for those with family histories of cancer could potentially have a significant impact on increasing screening rates.

The significant positive correlations between knowledge of cancer and knowledge about cancer screening (*r* = 0.262, *p* < 0.001) and between knowledge of cancer and attitudes towards screening (*r* = 0.236, *p* < 0.001) suggest a synergistic relationship among these components. This aligns with the IMB model’s conceptualization of information and motivation as interrelated constructs (44). However, the lack of significant correlations among other latent variables indicates that the relationships within the IMB model may be more complex in the UAE context than in other populations studied. This complexity highlights the need for nuanced, culturally sensitive approaches to promoting cancer screening in the UAE.

The inverse association observed between behavioral skills and cancer screening intention contrasts with the theoretical assumptions of the IMB model, in which behavioral skills are generally expected to facilitate health-related behavioral intentions and subsequent action. Several methodological and contextual explanations may account for this finding. This finding may reflect the poor model fit and exploratory nature of the analysis, rather than a true inverse relationship. It may also be influenced by contextual factors, such as perceived risk, family history, perceived barriers, and healthcare exposure. Furthermore, the single dichotomous measure of screening intention may have limited the sensitivity of the outcome. Therefore, this finding should be interpreted cautiously and requires replication using validated multi-item measures and representative samples.

First, the overall model demonstrated relatively poor fit, and the analysis was exploratory; therefore, the unexpected coefficient may reflect model misspecification or instability in the estimated relationships rather than a true inverse association. Second, behavioral skills in the present study were assessed using items related to self-efficacy and objective ability to undertake cancer screening. These skills may not necessarily translate directly into intention when other motivational or contextual factors, such as perceived risk, family experience, fear, perceived benefits and barriers, or access to screening services, are influential. Third, the broad, non-specific wording of the screening intention item may have introduced heterogeneity in participant responses. Some respondents may have visualized highly invasive procedures (e.g., colonoscopy) when responding, while others may have considered low-burden blood tests. If participants with stronger behavioral skills also had more accurate knowledge of the procedural demands of specific cancer screening tests, they might have responded less favorably to an unspecific screening item compared to those with less awareness. This could partially explain the inverse association observed and underscores the need for modality-specific intention measures in future investigations. Finally, the healthcare-based convenience sample may have included individuals with different levels of prior healthcare exposure and screening experience compared with the general UAE population. Accordingly, the observed inverse relationship should not be interpreted as evidence that greater behavioral skills reduce screening intention. Rather, it represents an exploratory finding that requires replication using validated multi-item measures, larger representative samples, and theoretically prespecified models.

These findings have several implications for public health practice and policy in the UAE. First, there is a clear need for tailored educational interventions that address specific knowledge gaps, particularly regarding less recognized symptoms such as unexplained lumps, and risk factors such as family history. Given that knowledge variables were not significantly associated with behavioral skills, these educational efforts should be integrated with motivational strategies rather than delivered in isolation. Second, addressing logistical barriers, such as transportation difficulties (factor loading 0.862), should be a priority. This could involve developing mobile screening units or transportation assistance programs, similar to initiatives that have shown success in other countries with geographically dispersed populations, such as Australia’s mobile screening programs for rural areas (45). Third, the strong influence of family history and perceived risk on screening intentions suggests that risk-based screening promotion could be highly effective. This approach has shown success in contexts such as the United Kingdom’s risk-stratified screening programs (46) and could be adapted to the UAE context. Fourth, the non-significant mediation findings suggest that interventions should focus on direct pathways to screening intention—particularly family history communication, risk perception enhancement, and addressing logistical barriers—rather than assuming that knowledge will automatically translate to skills and then to behavior. Techniques such as implementation intentions, which have shown effectiveness in bridging the intention-behavior gap in health contexts (47), could be particularly useful for translating positive attitudes into actual screening behavior. Future research should explore the cultural factors that may mediate the relationships between IMB components in the UAE context, similar to studies conducted in other Middle Eastern countries (48).

## 6 Strengths and limitations

This study contributes valuable evidence regarding cancer screening intentions among adults in the United Arab Emirates through the application of the Information–Motivation–Behavioral Skills (IMB) model. Several methodological strengths enhance the credibility of the findings. First, the study adopted a robust theoretical framework that integrates cognitive, motivational, and behavioral determinants of health behavior, allowing for a comprehensive examination of factors influencing cancer screening intentions. The questionnaire demonstrated strong psychometric properties, including excellent content validity and reliability, while the rigorous translation and back-translation procedures ensured cultural and linguistic appropriateness for both Arabic- and English-speaking participants. Furthermore, the use of advanced statistical techniques, including confirmatory factor analysis and latent variable structural equation modeling, enabled the assessment of complex relationships among latent constructs and provided a deeper understanding of the mechanisms underlying screening intentions. The inclusion of psychosocial factors such as perceived cancer risk, family history, and participation in awareness activities further strengthened the study by offering a multidimensional perspective. Importantly, the research addresses a significant knowledge gap in the UAE context, particularly within Ras Al Khaimah, generating evidence that may support the development of targeted cancer prevention and screening initiatives.

Despite these strengths, several limitations should be acknowledged when interpreting the findings. Most notably, the structural model demonstrated only marginal fit to the data, suggesting that the IMB framework, as operationalized in this study, may not fully explain cancer screening intentions within this cultural context and that the findings should be considered exploratory. Furthermore, the assessment of screening intention using a single dichotomous item may not fully capture the complexity and strength of behavioral intentions. The structural equation model demonstrated only marginal fit, indicating that the findings should be interpreted as exploratory and warrant further validation. The mediation analysis did not incorporate bootstrap confidence intervals for the indirect effects. Although indirect effects were evaluated using their estimated standard errors and *p*-values, the absence of bootstrap confidence intervals limits the robustness of inference regarding the hypothesized mediation pathways. Future studies should employ bootstrapped indirect-effect estimates with confidence intervals to provide more robust evidence regarding mediation.

Future research should employ longitudinal designs to examine the progression from screening intention to actual screening behavior and to establish temporal relationships among the identified predictors. Nationally representative studies using probability sampling across all emirates are needed to improve generalizability and provide a more comprehensive understanding of cancer screening behaviors in the UAE. Researchers should also utilize multi-item measures of screening intention to enhance reliability and capture the multidimensional nature of behavioral intentions. Qualitative investigations, including interviews and focus groups, are recommended to explore cultural, social, and contextual factors that may explain the observed relationships and the non-significant mediation effects identified within the Information–Motivation–Behavioral Skills model. Finally, future studies should evaluate alternative or adapted theoretical frameworks that incorporate cultural, socioeconomic, and healthcare system factors to better explain cancer screening behavior within the UAE context.

## 7 Implications for practice, policy, and future research

The findings of this study provide important implications for healthcare practice, public health policy, and future research aimed at improving cancer screening uptake in the United Arab Emirates. The strong influence of family history of cancer and perceived personal risk on screening intentions suggests that personalized risk communication strategies may be more effective than generalized awareness campaigns. Healthcare professionals should routinely assess family cancer history and individual risk perceptions during clinical encounters and use tailored counseling approaches to encourage participation in cancer screening programs. Risk-based communication may enhance motivation and strengthen individuals’ intentions to engage in preventive health behaviors.

The study further highlights the importance of addressing attitudinal and behavioral barriers to screening. Interventions should extend beyond knowledge dissemination and focus on enhancing self-efficacy, reducing fear and misconceptions, and addressing practical concerns related to screening procedures. Public health campaigns should incorporate behavioral support components, culturally sensitive messaging, and patient navigation services to facilitate access to screening services. Given the observed importance of logistical barriers, policymakers should consider strategies that improve accessibility, including transportation support, flexible appointment scheduling, workplace screening initiatives, and expanded community-based screening programs.

From a policy perspective, the findings support the development of targeted national cancer prevention and screening strategies that integrate individual, familial, and contextual determinants of screening behavior. Particular attention should be directed toward high-risk populations and groups with limited access to healthcare resources. Strengthening community awareness programs and integrating cancer risk assessment into primary healthcare services may enhance early detection efforts and contribute to improved cancer outcomes across the UAE.

## 8 Conclusion

This study provides novel evidence on the factors influencing cancer screening intentions among adults in the United Arab Emirates through the application of the Information–Motivation–Behavioral Skills (IMB) framework. The findings demonstrate that perceived personal cancer risk, family history of cancer, and participation in cancer awareness activities may represent a potential avenue for supporting screening intentions; however, their effectiveness in increasing actual screening uptake should be evaluated through appropriately designed intervention studies. While positive attitudes toward screening were associated with stronger behavioral skills, knowledge alone did not significantly predict behavioral readiness, suggesting that increasing awareness without addressing motivational and contextual factors may have limited impact on screening uptake. Notably, the hypothesized mediating role of behavioral skills was not supported, and the overall model exhibited only modest explanatory performance, indicating that conventional IMB pathways may not fully capture the complex sociocultural and healthcare-related determinants of cancer screening behavior within the UAE context. These findings underscore the need for more culturally responsive and context-specific approaches to understanding and promoting cancer screening.

From a public health perspective, the results emphasize the value of family-centered risk communication, targeted awareness programs, and interventions designed to reduce practical barriers to screening participation. Strengthening these components may enhance engagement with early detection services and contribute to improved cancer outcomes. Despite its exploratory nature, this study advances the understanding of cancer screening behavior in the UAE and provides an important foundation for future research, policy development, and evidence-based strategies aimed at increasing screening participation and reducing the burden of cancer through early detection.

## Data Availability

The raw data supporting the conclusions of this article will be made available by the authors, without undue reservation.

## References

[1] Ghufran MS Soni P Duddukuri GR. "The global concern for cancer emergence and its prevention: a systematic unveiling of the present scenario". In: Bioprospecting of Tropical Medicinal Plants. Cham: Springer Nature Switzerland (2023). p. 1429–55.

[2] Khokhar A Sharma P. "Role of environmental factors in increased Cancer incidences and health impacts". In: Biotechnological Innovations for Environmental Bioremediation. Singapore: Springer Nature Singapore (2022). p. 671–723.

[3] Sung H Ferlay J Siegel RL Laversanne M Soerjomataram I Jemal A . Global Cancer statistics 2020: GLOBOCAN estimates of incidence and mortality worldwide for 36 cancers in 185 countries. CA Cancer J Clin. (2021) 71:209–49. doi: 10.3322/caac.21660, 33538338

[4] Loney T Aw TC Handysides DG Ali R Blair I Grivna M . An analysis of the health status of the United Arab Emirates: the ‘big 4’ public health issues. Glob Health Action. (2013) 6:20100. doi: 10.3402/gha.v6i0.20100, 23394856 PMC3566378

[5] Radwan H Hasan H Ballout RA Rizk R. The epidemiology of cancer in the United Arab Emirates. Medicine. (2018) 97:e13618. doi: 10.1097/MD.0000000000013618, 30558043 PMC6320101

[6] Al-Shamsi HO Abyad AM. "A proposal for cancer control plan in the UAE". In: Cancer Care in the United Arab Emirates. Singapore: Springer Nature Singapore (2024). p. 91–126.

[7] Yasin JA Tamimi MA Qtaishat FA Al-Din TJ Obeidat DM Alkuttob LA . Lung cancer screening health belief model: psychometric properties of the Arabic version and factors influencing screening and prevention among Jordanians. Psychooncology. (2024) 33. doi: 10.1002/pon.70019, 39510976

[8] AlShamlan NA AlOmar RS AlAbdulKader AM AlGhamdi FA Aldakheel AA Al Shehri SA . Beliefs and utilization of cervical cancer screening by female health care workers in Saudi Arabia using the health belief model: a nationwide study. Int J Women's Health. (2023) 15:1245–59. doi: 10.2147/IJWH.S415924 PMC10417788 37576181

[9] Kamyab A Mohammadkhah F Asadi S Ghalehgolab F Khani Jeihooni A. The effect of an educational intervention on breast cancer screening of rural women: application of the theory of planned behavior. Cancer Control. (2024) 31. doi: 10.1177/10732748241266788, 39052977 PMC11282563

[10] Mirzaei-Alavijeh M Amini M Moradinazar M Eivazi M Jalilian F. Disparity in cognitive factors related to cancer screening uptake based on the theory of planned behavior. BMC Cancer. (2024) 24:845. doi: 10.1186/s12885-024-12607-w, 39014335 PMC11251123

[11] Chang SJ Choi S Kim SA Song M. Intervention strategies based on information-motivation-behavioral skills model for health behavior change: a systematic review. Asian Nurs Res (Korean Soc Nurs Sci). (2014) 8:172–81. doi: 10.1016/j.anr.2014.08.002, 42574925

[12] Ghasemi M Savabi-Esfahani M Noroozi M Satari M. Prediction of cervical cancer screening: application of the information-motivation-behavioral skills model. BMC Cancer. (2024) 24:351. doi: 10.1186/s12885-024-12098-9, 38504202 PMC10949627

[13] Johnson EM Ross DB. The cost of cancer: the association of financial and cancer-related stress on maladaptive coping styles in families with a cancer diagnosis. Contemp Fam Ther. (2023) 45:323–32. doi: 10.1007/s10591-021-09595-5, 34305314 PMC8290390

[14] Naito Y Nishida T Doi T. Current status of and future prospects for the treatment of unresectable or metastatic gastrointestinal stromal tumours. Gastric Cancer. (2023) 26:339–51. doi: 10.1007/s10120-023-01381-6, 36913072 PMC10115693

[15] Ward ZJ Scott AM Hricak H Atun R. Global costs, health benefits, and economic benefits of scaling up treatment and imaging modalities for survival of 11 cancers: a simulation-based analysis. Lancet Oncol. (2021) 22:341–50. doi: 10.1016/S1470-2045(20)30750-6, 33662286 PMC8033570

[16] Hanna TP King WD Thibodeau S Jalink M Paulin GA Harvey-Jones E . Mortality due to cancer treatment delay: systematic review and meta-analysis. BMJ. (2020) 371:m4087. doi: 10.1136/bmj.m4087, 33148535 PMC7610021

[17] Fisher JD Fisher WA Misovich SJ Kimble DL Malloy TE. Changing AIDS risk behavior: effects of an intervention emphasizing AIDS risk reduction information, motivation, and behavioral skills in a college student population. Health Psychol. (1996) 15:114–23. doi: 10.1037/0278-6133.15.2.114, 8681919

[18] Wang H Chang R Shen Q Tsamlag L Zhang S Shi Y . Information-motivation-behavioral skills model of consistent condom use among transgender women in Shenyang, China. BMC Public Health. (2020) 20:394. doi: 10.1186/s12889-020-08494-y, 32216775 PMC7098100

[19] Robinson WT. Adaptation of the information-motivation-behavioral skills model to needle sharing behaviors and hepatitis C risk. SAGE Open. (2017) 7. doi: 10.1177/2158244016666126

[20] Wells A Allen-Brown V Alam N Skulski C Jackson AL Herzog TJ. The importance of information, motivation, and behavioral skills (IMB): healthcare provider perspectives on improving adherence to cervical cancer screening among at-risk women. Public Health Pract. (2021) 2:100079. doi: 10.1016/j.puhip.2021.100079, 36101626 PMC9461580

[21] Khademian Z Zahmatkeshan N Rakhshan M Zarshenas L. The effectiveness of an intervention based on the information, motivation, and behavioral skills model on treatment adherence and self-efficacy in patients with coronary artery diseases: an explanatory mixed method study. J Iran Med Counc. (2024). doi: 10.18502/jimc.v7i4.16633

[22] Yao X Zhang L Du J Gao L. Effect of information-motivation-behavioral model based on protection motivation theory on the psychological resilience and quality of life of patients with type 2 DM. Psychiatry Q. (2021) 92:49–62. doi: 10.1007/s11126-020-09783-w, 32445004

[23] Fisher WA Fisher JD Harman J. "The information-motivation-behavioral skills model: a general social psychological approach to understanding and promoting health behavior". In: Social Psychological Foundations of Health and Illness. Wiley (2003). p. 82–106.

[24] Kline RB. Principles and Practice of Structural Equation Modeling. 4th ed. New York: Guilford Publications (2015).

[25] Siddiqui K. Heuristics for sample size determination in multivariate statistical techniques. World Appl Sci J. (2021) 27:285–7. doi: 10.5829/idosi.wasj.2013.27.02.889

[26] Al-Azri M Al-Hamedi I Al-Awisi H Al-Hinai M Davidson R. Public awareness of warning signs and symptoms of cancer in Oman: a community-based survey of adults. Asian Pac J Cancer Prev. (2015) 16:2731–7. doi: 10.7314/APJCP.2015.16.7.2731, 25854355

[27] Kim BK Jo HS Lee HJ. Study on the factors related with intention of cancer screening among Korean residents. Asia Pac J Public Health. (2015) 27:NP2133–43. doi: 10.1177/1010539511422374, 22234830

[28] Hair J Hult GTM Ringle C Sarstedt M. A primer on partial least squares structural equation modeling (PLS-SEM). Interne. (2022). doi: 10.1007/978-3-030-80519-7, 30311153

[29] Shmueli G Sarstedt M Hair JF Cheah JH Ting H Vaithilingam S . Predictive model assessment in PLS-SEM: guidelines for using PLSpredict. Eur J Mark. (2019) 53:2322–47. doi: 10.1108/EJM-02-2019-0189

[30] Singhai S Singh R Sardana HK Madhukar A. Analysis of factors influencing technology transfer: a structural equation modeling based approach. Sustainability (Basel). (2021) 13:5600. doi: 10.3390/su13105600

[31] Ferguson SL Moore G Ew Hull DM. Finding latent groups in observed data: a primer on latent profile analysis in Mplus for applied researchers. Int J Behav Dev. (2020) 44:458–68. doi: 10.1177/0165025419881721

[32] Hong S Lee YY Lee J Kim Y Choi KS Jun JK . Trends in Cancer screening rates among Korean men and women: results of the Korean National Cancer Screening Survey, 2004–2018. Cancer Res Treat. (2021) 53:330–8. doi: 10.4143/crt.2020.263, 33091969 PMC8053859

[33] Schliemann D Ismail R Donnelly M Cardwell CR Su TT. Cancer symptom and risk factor awareness in Malaysia: findings from a nationwide cross-sectional study. BMC Public Health. (2020) 20:464. doi: 10.1186/s12889-020-08581-0, 32252721 PMC7137199

[34] Al-Azri M Al-Maskari A Al-Matroushi S Al-Awisi H Davidson R Panchatcharam SM . Awareness of cancer symptoms and barriers to seeking medical help among adult people attending primary care settings in Oman. Health Serv Res Manag Epidemiol. (2016) 3. doi: 10.1177/2333392816673290, 28462284 PMC5266467

[35] Elobaid YE Aw TC Grivna M Nagelkerke N. Breast Cancer screening awareness, knowledge, and practice among Arab women in the United Arab Emirates: a cross-sectional survey. PLoS One. (2014) 9:e105783. doi: 10.1371/journal.pone.0105783, 25265385 PMC4179300

[36] Sheshachalam A Chakravarthy A. The cancer awareness assessment project: a small-scale survey across people with different levels of education in Mysore, India. Indian J Cancer. (2015) 52:153–5. doi: 10.4103/0019-509X.175574, 26838007

[37] Wang Z Wang Z Wang Y Wu J Yu Z Chen C . High risk and early onset of cancer in Chinese patients with Peutz-Jeghers syndrome. Front Oncol. (2022) 12. doi: 10.3389/fonc.2022.900516, 36033506 PMC9406140

[38] Alsowiyan A Almotyri H Alolayan N Alissa L Almotyri B AlSaigh S. Breast cancer knowledge and awareness among females in Al-Qassim region, Saudi Arabia in 2018. J Family Med Prim Care. (2020) 9:1712–8. doi: 10.4103/jfmpc.jfmpc_1065_19, 32509677 PMC7266218

[39] Hallgren E Yeary KHK DelNero P Johnson-Wells B Purvis RS Moore R . Barriers, facilitators, and priority needs related to cancer prevention, control, and research in rural, persistent poverty areas. Cancer Causes Control. (2023) 34:1145–55. doi: 10.1007/s10552-023-01756-1, 37526781 PMC10547626

[40] Elmaghraby DA Alshalla AA Alyahyan A Altaweel M Al ben Hamad AM Alhunfoosh KM . Public knowledge, practice, and attitude regarding cancer screening: a community-based study in Saudi Arabia. Int J Environ Res Public Health. (2023) 20:1114. doi: 10.3390/ijerph20021114, 36673870 PMC9859105

[41] Howell D Mayer DK Fielding R Eicher M Verdonck-de Leeuw IM Johansen C . Management of Cancer and Health after the clinic visit: a call to action for self-Management in Cancer Care. JNCI J Natl Cancer Inst. (2021) 113:523–31. doi: 10.1093/jnci/djaa083, 32525530 PMC8096367

[42] Karl JA Fischer R Druică E Musso F Stan A. Testing the effectiveness of the health belief model in predicting preventive behavior during the COVID-19 pandemic: the case of Romania and Italy. Front Psychol. (2022) 12:12. doi: 10.3389/fpsyg.2021.627575, 35095628 PMC8789680

[43] Feng GC Lin Z Ou W Su X Yan Q. A model-based meta-analysis of willingness to participate in Cancer screening. Int J Environ Res Public Health. (2021) 18:2580. doi: 10.3390/ijerph18052580, 33806652 PMC7967393

[44] John SA Walsh JL Weinhardt LS. The information–motivation–behavioral skills model revisited: a network-perspective structural equation model within a public sexually transmitted infection clinic sample of hazardous alcohol users. AIDS Behav. (2017) 21:1208–18. doi: 10.1007/s10461-016-1446-2, 27260181 PMC5136346

[45] Dodd RH Sharman AR Yap ML Stone E Marshall H Rhee J . “We need to work towards it, whatever it takes.”—participation factors in the acceptability and feasibility of lung cancer screening in Australia: the perspectives of key stakeholders. Transl Lung Cancer Res. (2024) 13:240–55. doi: 10.21037/tlcr-23-623, 38496699 PMC10938089

[46] McWilliams L Evans DG Payne K Harrison F Howell A Howell SJ . Implementing risk-stratified breast screening in England: an agenda setting meeting. Cancers (Basel). (2022) 14:4636. doi: 10.3390/cancers14194636, 36230559 PMC9563640

[47] Fennis BM Adriaanse MA Stroebe W Pol B. Bridging the intention–behavior gap: inducing implementation intentions through persuasive appeals. J Consum Psychol. (2011) 21:302–11. doi: 10.1016/j.jcps.2010.12.003

[48] Benamer HT Bakoush O. Arab nations lagging behind other middle eastern countries in biomedical research: a comparative study. BMC Med Res Methodol. (2009) 9:26. doi: 10.1186/1471-2288-9-26, 19374747 PMC2674457

